# A Real-Time Mobile Intervention to Reduce Sedentary Behavior Before and After Cancer Surgery: Pilot Randomized Controlled Trial

**DOI:** 10.2196/41425

**Published:** 2023-01-12

**Authors:** Carissa A Low, Michaela Danko, Krina C Durica, Julio Vega, Meng Li, Abhineeth Reddy Kunta, Raghu Mulukutla, Yiyi Ren, Susan M Sereika, David L Bartlett, Dana H Bovbjerg, Anind K Dey, John M Jakicic

**Affiliations:** 1 University of Pittsburgh Pittsburgh, PA United States; 2 University of Washington Seattle, WA United States; 3 Allegheny Health Network Cancer Institute Pittsburgh, PA United States; 4 University of Kansas Medical Center Kansas City, KS United States

**Keywords:** sedentary behavior, mobile health, smartphone, mobile phone, wearable device, surgical oncology, physical activity, perioperative cancer patients, surgical recovery, abdominal cancer surgery, perioperative intervention, activity monitoring

## Abstract

**Background:**

Sedentary behavior (SB) is prevalent after abdominal cancer surgery, and interventions targeting perioperative SB could improve postoperative recovery and outcomes. We conducted a pilot study to evaluate the feasibility and preliminary effects of a real-time mobile intervention that detects and disrupts prolonged SB before and after cancer surgery, relative to a monitoring-only control condition.

**Objective:**

Our aim was to evaluate the feasibility and preliminary effects of a perioperative SB intervention on objective activity behavior, patient-reported quality of life and symptoms, and 30-day readmissions.

**Methods:**

Patients scheduled for surgery for metastatic gastrointestinal cancer (n=26) were enrolled and randomized to receive either the SB intervention or activity monitoring only. Both groups used a Fitbit smartwatch and companion smartphone app to rate daily symptoms and collect continuous objective activity behavior data starting from at least 10 days before surgery through 30 days post discharge. Participants in the intervention group also received prompts to walk after any SB bout that exceeded a prespecified threshold, with less frequent prompts on days that patients reported more severe symptoms. Participants completed end-of-study ratings of acceptability, and we also examined adherence to assessments and to walking prompts. In addition, we examined effects of the intervention on objective SB and step counts, patient-reported quality of life and depressive and physical symptoms, as well as readmissions.

**Results:**

Accrual (74%), retention (88%), and acceptability ratings (mean overall satisfaction 88.5/100, SD 9.1) were relatively high. However, adherence to assessments and engagement with the SB intervention decreased significantly after surgery and did not recover to preoperative levels after postoperative discharge. All participants exhibited significant increases in SB and symptoms and decreases in steps and quality of life after surgery, and participants randomized to the SB intervention unexpectedly had longer maximum SB bouts relative to the control group. No significant benefits of the intervention with regard to activity, quality of life, symptoms, or readmission were observed.

**Conclusions:**

Perioperative patients with metastatic gastrointestinal cancer were interested in a real-time SB intervention and rated the intervention as highly acceptable, but engagement with the intervention and with daily symptom and activity monitoring decreased significantly after surgery. There were no significant effects of the intervention on step counts, patient-reported quality of life or symptoms, and postoperative readmissions, and there was an apparent adverse effect on maximum SB. Results highlight the need for additional work to modify the intervention to make reducing SB and engaging with mobile health technology after abdominal cancer surgery more feasible and beneficial.

**Trial Registration:**

ClinicalTrials.gov NCT03211806; https://tinyurl.com/3napwkkt

## Introduction

Surgical treatment is a critical component of curative therapy for most cancers, but risks for postoperative complications, unplanned readmissions, and persistent functional impairments are common, especially for abdominal cancers, where rates of complications and readmissions can range from 25%-50% [1-3]. These high rates of adverse postoperative outcomes place patients at risk for functional limitations and impaired quality of life as well as high health care costs and utilization. Supportive interventions aimed at optimizing perioperative health and functioning are needed for this high-risk surgical oncology population.

Physical activity is one modifiable behavior that holds promise for affecting postoperative recovery and outcomes [4-6]. Indeed, prehabilitation programs that promote physical activity before surgery have been linked to improved preoperative functional capacity [7] and shorter length of stay after cancer surgery [8]. Similarly, early mobilization after surgery, generally defined as out of bed activity by postoperative day one, is recommended as part of Enhanced Recovery after Surgery pathways, although evidence of benefit is mixed [9]. Because both prehabilitation and Enhanced Recovery after Surgery often include nutritional interventions and other components, it is difficult to determine whether and to what extent increased physical activity alone can reduce postoperative risks. Moreover, postoperative symptoms such as pain and fatigue make increasing physical activity after cancer surgery challenging and may compromise adherence to exercise interventions [10,11]. In the perioperative surgical oncology context, interventions aimed at disrupting prolonged sedentary behavior (SB) with brief walking breaks may be more attainable than more structured exercise interventions, especially if the intervention can adapt to changing symptom burden over the perioperative course. To date, no studies have tested the impact of perioperative SB disruption on surgical oncology outcomes [12].

The goal of this study was to pilot-test a personalized mobile technology–supported intervention to reduce SB before and after gastrointestinal cancer surgery. This intervention uses a smartwatch and smartphone to collect daily symptom ratings that are used to tailor the frequency of prompts to disrupt prolonged SB in real time, which we hypothesized would increase the feasibility of the intervention. We previously described the development and usability of this intervention in a single-arm pilot trial [13]. In this pilot randomized controlled trial, we compared patients randomized to receive the SB intervention to those whose activity and symptoms were monitored only. The primary outcome of this initial pilot trial was feasibility, defined as accrual and retention, end-of-study acceptability ratings, and adherence to intervention assessments and activity prompts. Secondary outcomes included objective activity and SB, patient-reported quality of life and symptoms, as well as postoperative readmissions.

## Methods

### Participants

Participants were recruited from the outpatient clinics of 6 surgeons specializing in abdominal cancer surgery at a National Cancer Institute–designated comprehensive cancer center. Participants were recruited between June 2019 and March 2021 at their preoperative surgical oncology clinic visit. Study accrual was paused from March to December of 2020 due to the COVID-19 pandemic. Research staff provided study information and email reminders to the 6 surgical oncology care teams and asked the nurse or physician assistant to identify potential patients at the time of their consent to surgery, confirm their eligibility, and to either consent them directly or connect them with the research team for consent and onboarding. The study was open to English-speaking adults scheduled for surgical treatment of metastatic gastrointestinal or peritoneal cancer and able to stand and walk unassisted. Exclusion criteria included having less than 10 days to scheduled surgery date, to provide adequate time for participants to become familiar with study technology and activity prompts prior to surgery. No participants had sensory or motor impairments that interfered with use of the study apps.

### Study Procedures

Following completion of written informed consent, participants were randomized via random number generator to either the SB intervention (which included activity monitoring) or activity monitoring only. They were provided with a Fitbit Versa smartwatch (first generation) paired with a Google Pixel 2 smartphone on which Detecting Activity to Support Healing (DASH) study apps (Intervention or Monitoring-only) as well as the Fitbit app had been installed. From the time of consent to 30 days after hospital discharge following their surgery, participants were asked to keep the devices charged, to wear the smartwatch as much as possible, to rate their daily experience of symptom severity once each morning, and for intervention participants only, to respond to activity prompts. Participants completed a questionnaire at study entry to collect information about demographic variables, health behaviors, and experience with mobile technology. Before surgery, during inpatient recovery, and approximately 30 days after postoperative discharge, participants also completed standardized measures of depressive and physical symptoms and quality of life. At the end of the study, all participants completed a semistructured interview about their experiences with the devices and a questionnaire about the acceptability and usability of the apps.

As previously described [13], all participants used the DASH Android smartphone study app to rate the daily severity of 10 symptoms (ie, pain, fatigue or tiredness, sleep disturbance, trouble concentrating or remembering things, feeling sad or down, feeling anxious or worried, shortness of breath, numbness or tingling, nausea, and diarrhea or constipation), using a scale from 0 (ie, symptom not present) to 10 (ie, symptom as bad as you can imagine). Participants were randomized to either the DASH intervention or monitoring-only control condition. Participants randomized to the DASH intervention received a Fitbit smartwatch app that used the most recent symptom rating to set a threshold for SB bouts and used real-time step count data to trigger activity prompt notifications when prespecified SB thresholds were exceeded (60 consecutive minutes of SB when all symptoms were rated less than 7 out of 10 or 120 consecutive minutes of SB if any symptom was rated as 7 or higher). For the purposes of this study, SB was operationalized as a minute with fewer than 10 steps logged by the Fitbit, to allow for incidental stepping and arm movements that might be misclassified as steps, while also classifying very slow walking as activity, given the perioperative context and likely diminished gait cadence during early postoperative recovery [14]. When SB thresholds were exceeded, an activity prompt (“Ready for a short walk?”) was sent to the smartwatch. If 30 or more steps were logged within 15 minutes of an activity prompt, participants received a positive feedback message (“Great job being active!”). Prompts were sent only between each participant’s waking time and bedtime, which were set by participants in the Android app and could be adjusted during the study. Participants randomized to monitoring-only received a Fitbit smartwatch app that measured steps but did not send activity prompts.

### Ethics Approval

All procedures were approved by the University of Pittsburgh Institutional Review Board (STUDY 19030389) and registered on ClinicalTrials.gov (NCT03211806).

### Measures

Primary outcome measures assessing feasibility were (1) accrual and retention rates (ie, percentage of participants approached who enrolled in the research and percentage of participants enrolled who completed the study); (2) acceptability, based on end-of-study responses to the System Usability Scale [15] and the following questions: “On a scale of 0-100, how easy was it to use the smartphone/watch?” “On a scale of 0-100, how pleasant was the smartphone/watch interface (appearance, design, usability)?” and “On a scale of 0-100, how satisfied were you with the overall system (including the smartphone and watch and all notifications)?”; as well as (3) adherence (percentage of days symptom ratings were completed and at least 8 hours of Fitbit data were logged, and for intervention participants only, percentage of activity prompts after which steps were detected).

Secondary outcome measures included (1) objective SB (maximum and mean SB bout duration per day based on Fitbit minutes with less than 10 steps logged); (2) objective physical activity (Fitbit step count per day); (3) patient-reported symptoms (depressive symptoms via Center for Epidemiological Studies-Depression [16]) and physical symptoms via questions adapted from the MD Anderson Symptom Inventory and based on the National Cancer Institute’s Symptom Management and Health-Related Quality of Life Steering Committee recommendations [17,18]; (4) patient-reported quality of life via the Functional Assessment of Cancer Therapy [19]; and (5) readmissions within 30 days after index hospital discharge, extracted from electronic medical records.

### Analytic Approach

Group differences in baseline participant characteristics and end-of-study acceptance and usability ratings were examined using independent sample two-tailed *t* tests and chi-squared tests. Linear mixed modeling assuming the best-fitting variance-covariance structure for the repeated assessments was used to explore the effect of the intervention over the 3 study time points (ie, preoperative, inpatient, and after discharge) for the outcomes of adherence, SB, physical activity, psychological and physical symptoms, and quality of life. The models included a fixed, between-subjects effect for randomized group assignment as well as a fixed, within-subject effect for time and group interaction by time. In addition, to test statistics (*F* test values) and corresponding *P* values from the model, least square means with standard errors are presented. Two outcomes, average SB and steps, were square-root transformed due to positively skewed residual distributions when modeling the outcome in its original metric. Data for Fitbit step counts and SB bout duration were only included from days that the Fitbit was worn at least 8 hours, and sleep episodes as identified by the Fitbit were excluded from SB bouts.

## Results

### Participant Characteristics

As shown in Table 1, the sample was primarily White and predominantly male, with most patients undergoing cytoreductive surgery with hyperthermic intraperitoneal chemotherapy. Participants randomized to the intervention arm had significantly higher BMI and were less likely to be a former smoker compared to those randomized to the control arm. Participants started using the DASH apps a mean of 19.6 (range 8-47) days prior to surgery, throughout their inpatient stay as feasible (which lasted an average of 10.9, range 5-24 days), and for 30 days post discharge, for an average of 57.2 total days (range 44-92 days) of study participation.

**Table 1:** Participant characteristics.

Characteristics			All participants (n=26)	Intervention arm (n=13)		Monitoring-only arm (n=13)
Age (years), mean (SD)			56.2 (10.5)	54.9 (6.5)		57.5 (13.5)
**Sex, n (%)**						
	Female	11 (42.3)		7 (53.8)	4 (30.8)	
	Male	15 (57.7)		6 (46.2)	9 (69.2)	
**Race, n (%)**						
	White	24 (92.3)		13 (100)	11 (84.6)	
	Black	1 (3.8)		0 (0)	1 (7.7)	
	More than one	1 (3.8)		0 (0)	1 (7.7)	
BMI, mean (SD)			27.4 (5.3)	30.2 (5.8)		24.8 (3.3)
**Smoking history, n (%)**						
	Current smoker	1 (4)		1 (8.3)	0 (0)	
	Former smoker	5 (20)		0 (0)	5 (38.5)	
	Never a smoker	19 (76)		11 (91.7)	8 (61.5)	
**Exercise frequency, n (%)**						
	Seldom or never	6 (24)		4 (33.3)	2 (15.4)	
	1-2 times per week	7 (28)		5 (41.7)	2 (15.4)	
	3-4 times per week	10 (40)		3 (25)	7 (53.8)	
	>5 times per week	2 (8)		0 (0)	2 (15.4)	
Has Wi-Fi at home, n (%)			22 (88)	11 (91.7)		11 (84.6)
Owns a smartphone, n (%)			26 (100)	12 (100)		13 (100)
Owns an activity tracker, n (%)			4 (16)	2 (16.7)		2 (15.4)
CS+HIPEC^a^ surgery, n (%)			17 (65.4)	10 (76.9)		7 (53.8)

^a^CS+HIPEC: cytoreductive surgery with hyperthermic intraperitoneal chemotherapy.

### Primary Outcomes

#### Accrual and Retention

Of the 35 eligible patients approached, 26 consented to the study (74% accrual rate). Reasons for not participating were “too busy/overwhelmed” (n=2), “not good with technology” (n=3), “already wear a smartwatch/activity monitor and did not want to wear two” (n=2), and “had to leave clinic so did not have time to discuss the study” (n=2). The retention rate for the study was 88%, with 3 participants withdrawing (2 participants before starting to use the devices—one in intervention and one in monitoring-only condition—and 1 participant in the intervention condition 18 days after surgery due to poor health and readmission).

#### Acceptance

A total of 20 participants completed the end-of-study interview, and those in both the intervention and monitoring-only conditions rated the phone and watch interfaces as pleasant and easy to use and the overall system as satisfactory and usable (Table 2).

**Table 2:** Mean (SD) participant ratings of interface and system usability.

Variable (range 0-100)	All (n=20)	Intervention arm (n=9)	Monitoring-only arm (n=11)	*P* value
Phone—ease of use	93.1 (7.2)	91.1 (8.3)	94.6 (6.1)	.29
Watch—ease of use	91.6 (12.6)	94.4 (5.1)	89.3 (16.3)	.38
Phone—pleasantness	87.7 (13.7)	90.0 (13.2)	85.8 (14.5)	.51
Watch—pleasantness	87.9 (14.9)	91.7 (7.9)	84.8 (18.7)	.32
Overall satisfaction	88.5 (9.1)	91.3 (5.5)	86.2 (11.0)	.22
System Usability Scale	85.1 (11.5)	83.6 (9.2)	86.4 (13.5)	.61

When asked what they thought of the study, participants in the intervention condition reported that the activity prompts were motivating, especially prior to surgery. For example, one participant (P13) noted that “it got me moving more than I normally would have.” However, participants also noted that the prompts were not as motivating or as easy to respond to after surgery, especially in the hospital. One participant (P6) said the following:

At the beginning, I thought it was awesome and was very excited about the step counting and found it motivational; after surgery, I had a lot of trouble getting interest back, and the watch wasn't enough to be motivating; I lost interest because I had other priorities health-wise.

Another participant (P7) said the following:

It’s so much easier to get up presurgery. Maybe a hierarchy of prompts tapping different motivations [would be better] because it takes so much more to get up post-surgery.

Across both conditions, participants mentioned that they enjoyed tracking their step or sleep data in the Fitbit app. One participant (P16, in the monitoring-only group) noted the following:

Part of my recovery was setting step goals for myself and increasing that goal.

Some participants, like P2 (in the monitoring-only group), also noticed associations between activity and how they felt, noting “days with higher steps always felt better symptom-wise, looking back.” Many participants felt that physical activity was beneficial for their physical and psychological recovery, as P13 (in the intervention group) said the following:

Moving and walking helped prevent scar tissue development. If I had stayed sedentary, I think I would have been in much worse shape.

#### Adherence

**Figure 1:**
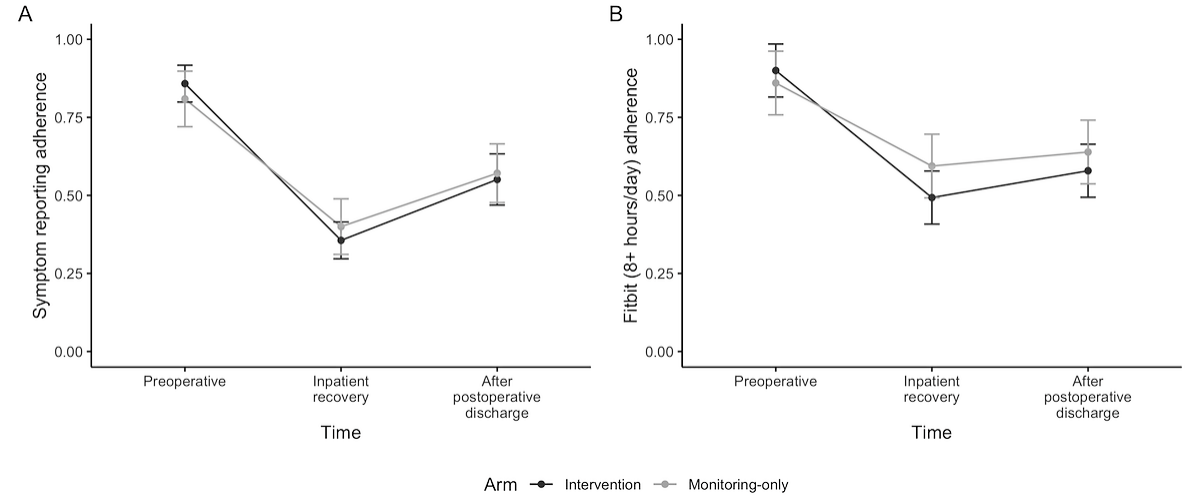
Proportion of days (in mean and SE) participants were adherent with (A) daily symptom reporting and (B) wearing the Fitbit for at least 8 hours per day.

Over the course of the study, daily symptom ratings were completed on 62% (874/1416) of days, ranging from 14% (9/65) to 95% (55/58) of days across individual participants. Fitbits were worn on 77% (1091/1416) of study days, and 91% (990/1091) of these days had at least 8 hours of Fitbit data available. On average, 69% (977/1416) of days were included in Fitbit analyses; across individual participants, the percentage of days with at least 8 hours of Fitbit data ranged from 17% (9/52 days with ≥8 hours of data) to 100% (58/58 days with ≥8 hours of data). As shown in Figure 1, participants became less adherent with both symptom reporting and wearing the Fitbit after surgery (symptom reporting: *F*_time_=22.9; *P*<.001; and Fitbit: *F*_time_=9.2; *P*=.001), but there were no significant differences in adherence between the two study groups (symptom reporting: *F*_group_=0.0; *P*=.95; *F*_group×time_=0.3; *P*=.78; and Fitbit: *F*_group_=0.2; *P*=.663; *F*_group×time_=0.4; *P*=.68).

For participants in the intervention group, an average of 5.8 activity prompts were sent per day, and participants took steps and received positive feedback after 22% (418/1925) of activity prompts. This varied substantially from before surgery (mean 3.3, SD 1.8 prompts per day; 200/407, 49% of prompts resulted in walking) to after surgery in the hospital (mean 7.8, SD 2.6 prompts per day; 29/462, 6% of prompts resulted in walking) to postdischarge recovery (mean 6.2, SD 2.6 prompts per day; 189/1056, 18% of prompts resulted in walking).

### Secondary Outcomes

#### Fitbit-Measured Sedentary Behavior Bouts and Steps

**Figure 2:**
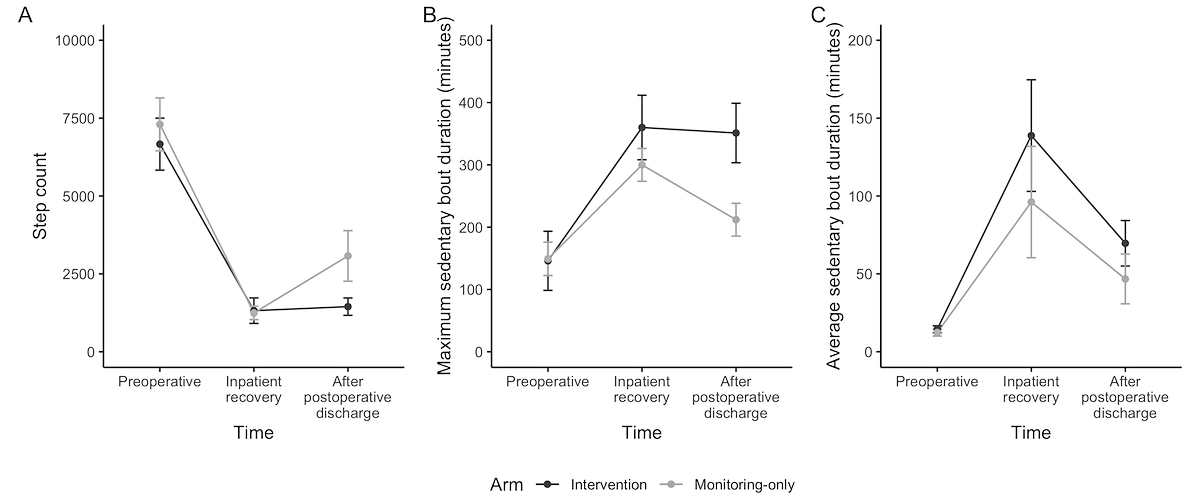
(A) Fitbit daily mean step count, (B) maximum sedentary bout duration, and (C) mean sedentary bout duration.

On average, participants logged 3642 (SD 3365) steps per day with a mean SB bout duration of 61 (SD 80) minutes and a maximum SB bout duration of 248 (SD 155) minutes. For all participants, step counts decreased significantly, and mean and maximum SB bout duration increased significantly from before surgery to during inpatient recovery (Figure 2; step count: *F*_time_=60.5; *P*<.001; maximum SB bout: *F*_time_=10.1; *P*<.001; and mean SB bout: *F*_time_=28.1; *P*<.001). The intervention had no significant effect on step count (*F*_group_=2.3; *P*=.15; and *F*_group×time_=1.2; *P*=.32) or mean SB bout duration (*F*_group_=1.5; *P*=.24; and *F*_group×time_=0.5; *P*=.24). Unexpectedly, participants randomized to the intervention had *longer* maximum SB bouts overall (*F*_group_=6.16; *P*=.02; and *F*_group×time_=1.48; *P*=.24). One important limitation to note is that these mean step count and SB bout values are based on the subset of participants who were compliant with wearing the smartwatch. Although there were no significant group differences in Fitbit compliance, intervention participants tended to wear the watch for fewer hours per day, and some mentioned removing the watch when they knew they would not be able to get up and walk or when trying to nap or rest. Because we included all days with at least 8 hours of total but not necessarily consecutive wear time, we may have misclassified some episodes during which participants were not wearing the watch as sedentary bouts. When hours of wear time per day was included in models, the group effect on maximum SB bout duration was no longer significant (data not shown).

#### Patient-Reported Measures

**Figure 3:**
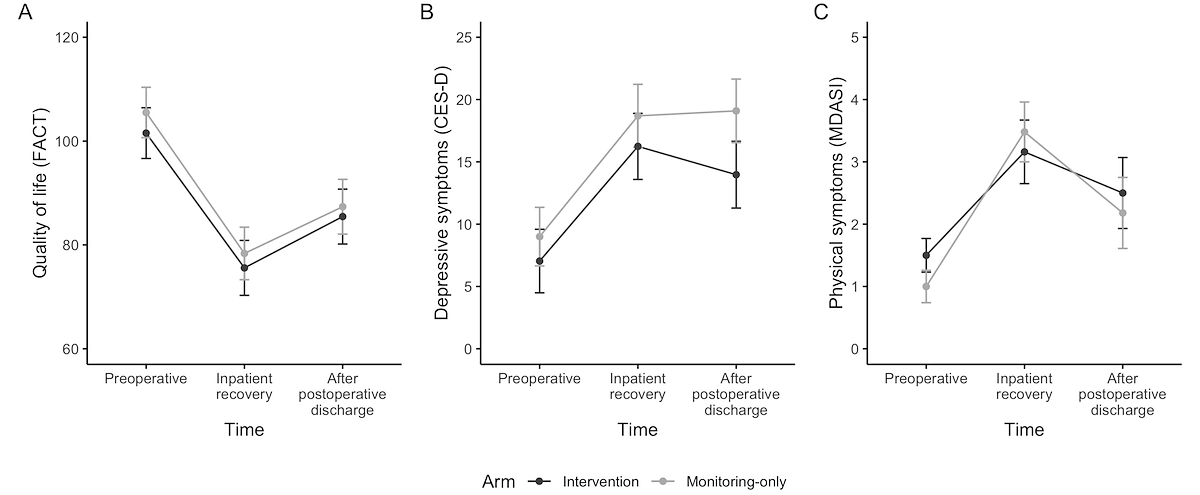
(A) Patient-reported quality of life, (B) depressive symptoms, and (C) physical symptoms. CES-D: Center for Epidemiological Studies-Depression; FACT: Functional Assessment of Cancer Therapy; MDASI: MD Anderson Symptom Inventory.

Similar to the other outcomes, we observed a significant time effect for quality of life (*F*_time_=21.4; *P*<.001; Figure 3), depressive symptoms (*F*_time_=10.9; *P*<.001), and physical symptoms (*F*_time_=24.0; *P*<.001), but no significant group (quality of life: *F*_group_=0.3; *P*=.60; depressive symptoms: *F*_group_=1.6; *P*=.22; and physical symptoms: *F*_group_=0.1; *P*=.76) or group × time effect (quality of life: *F*_group×time_=0.0; *P*=.97; depressive symptoms: *F*_group×time_=0.3; *P*=.78; and physical symptoms: *F*_group×time_=0.9; *P*=.41). All participants regardless of condition reported worsening quality of life and symptoms after surgery.

In total, 5 of 12 participants who started in the intervention condition were readmitted within 30 days, compared to 4 of 12 participants who started in the monitoring-only conditions, and there was no significant group difference in readmission rate (*χ*^1^_2_=0.2; *P*=.67).

## Discussion

### Principal Findings

In this paper, we described results from a pilot randomized trial testing a SB intervention in patients undergoing surgery for metastatic gastrointestinal cancer. To our knowledge, this is the first SB intervention developed specifically for patients undergoing cancer surgery at high risk for adverse postoperative outcomes [12]. Although patients were willing to participate and remain in the trial and rated the intervention as highly acceptable, engagement with the intervention and with daily symptom and activity monitoring decreased significantly after surgery. There were no significant effects of the intervention on step counts, patient-reported quality of life or symptoms, and postoperative readmissions. Contrary to hypotheses, participants randomized to the intervention group exhibited longer maximum SB bouts relative to the monitoring-only condition.

The SB intervention tested in this trial was designed to make replacing prolonged SB with short walking breaks more feasible in the perioperative context by reducing the frequency of SB prompts on days that patients reported high symptom burden. Given the significant drops in adherence and engagement that occurred after surgery and particularly during inpatient recovery as well as the fact that participants may have been less likely to complete symptom ratings on days they felt particularly unwell, additional modifications to the intervention are needed to make postoperative activity more feasible. A number of participants in the intervention condition noted that it was very difficult to get out of bed to walk when prompted, especially without assistance; this challenge and the associated frustration could have led to decreased self-efficacy to adhere to the intervention that carried into the postdischarge period, leading to increased SB and decreased adherence. Pausing the intervention until patients were recovering at home, adapting the intervention to involve caregivers or hospital staff and timing prompts around their availability to assist with postoperative ambulation, or replacing walking with light stretches or activities that could be done in bed while seated could all be options for future interventions targeting activity among postoperative inpatients. Adherence to assessments also decreased in the monitoring-only group after surgery, suggesting that either reduced frequency of assessments or additional support and reminders may be needed to make collection of continuous activity and daily symptom ratings feasible postoperatively.

The lack of observed benefits with regard to SB and activity may have been related to low adherence and engagement, or the intervention may not have been sufficiently robust to produce a change in activity during the acute perioperative period. Although participants in our usability and feasibility study reported that the frequency of prompts was appropriate, the current intervention was fairly minimal, aiming to disrupt SB bouts of 1-2 hours with a small (30 steps or more) amount of walking and positive reinforcement when goals were met. Participants may have disengaged from the intervention if they did not perceive it to be beneficial, and targeting a higher activity goal, if done in a feasible way that factors in physical limitations after surgery, could result in higher adherence over time if participants perceive the SB intervention to be meaningfully increasing activity and to have potential health benefits. In the future, providing education about the risks of perioperative SB, personalized goal setting to inform prompts, and coaching and problem-solving to overcome barriers to disrupting SB could be considered as additional interventional components to enhance a perioperative SB intervention [12,20]. Involving patients in the co-design of perioperative SB interventions could also result in enhanced engagement and benefit [21].

### Comparison to Prior Work

Although adherence to reporting symptoms and wearing the Fitbit declined significantly after surgery, rates were consistent with other work on wearables [22] and symptom reporting [23] during cancer treatment and with an earlier study of activity monitoring after cancer surgery [24]. As in our earlier field trial [13], adherence also varied substantially between participants, ranging from approximately 15% to 100% for both symptom reporting and Fitbit wearing throughout the perioperative period. Another approach to consider in future work is a more stepped-care approach, with more frequent contact or high-touch support for patients with poor adherence. Of note, because we used the same wearable device to both deliver the intervention and measure objective activity, poor adherence and engagement resulted in less accurate assessments of activity and SB, which may also have affected results, particularly if patients in the intervention group began wearing the device for fewer hours each day after surgery due to inability to respond to the walking prompts or to minimize disruptions caused by the prompts.

Although the intervention yielded no significant benefits for patients, this study highlights the continued need for interventions to improve postoperative recovery following surgery for metastatic abdominal cancer. Consistent with other studies, nearly 40% of patients in our sample experienced an unplanned hospital readmission, and participants remained significantly less active 30 days after postoperative discharge, relative to their preoperative activity levels. In addition to improved interventions targeting SB and activity, interventions aimed at remotely monitoring and addressing worsening pain or other symptoms as well as other causes of readmission (eg, dehydration) hold promise for their ability to support this high-risk population.

### Strengths and Limitations

Strengths of this study include the randomized design and the use of real-time symptom ratings and step data to trigger personalized just-in-time activity prompts. Focusing on disrupting SB rather than increasing physical activity is novel in the context of cancer surgery. Starting the intervention prior to scheduled surgery allowed participants to become familiar with the devices and begin increasing activity prior to surgery and hospitalization, while there may also be clinical value in continuing an intervention shortly after surgery when SB is very prevalent. The use of off-the-shelf consumer devices is also highly scalable.

This study had a number of important limitations. First, the sample size was smaller than originally intended due to COVID-19 pandemic–related disruptions to accrual and is an important limitation of this study. Second, developing a system capable of remotely detecting real-time step counts using a consumer wearable device proved challenging and required us to provide study Android phones to participants to use for collecting symptom ratings and synchronizing the Fitbit smartwatch. All enrolled participants already owned a personal smartphone; requiring them to also carry and charge an additional study smartphone across perioperative transitions of care may have contributed to adherence challenges; future interventions in this area should be deployed on participants’ existing phones to potentially improve feasibility. Third, we elected to use a monitoring-only control so that the only difference between the two study groups was the activity prompts in recognition of the fact that merely using an activity monitor can promote physical activity among cancer patients [25]; alternative control conditions could have yielded different results. Finally, all participants were undergoing surgery for metastatic peritoneal or gastrointestinal cancer, and results may not generalize to other perioperative groups or contexts.

### Future Directions

As described above, additional intervention refinement and testing is needed to make real-time SB disruption more feasible and engaging for an abdominal cancer surgery population, particularly during the postoperative period. Given the small sample in this work, larger trials of activity modification are necessary once the intervention has been improved to be more feasible and potentially more robust. There have been significant advances in consumer wearable technology since the DASH apps were developed in 2018, and future work should consider interventions that leverage Apple HealthKit or GoogleFit and work across different activity monitoring devices.

### Conclusions

In conclusion, although patients undergoing abdominal cancer surgery were interested in a real-time SB intervention and rated the intervention as highly acceptable, adherence and engagement decreased significantly after surgery, and there were no observed benefits of the intervention on objective activity, quality of life, symptoms, or readmissions. Further research may be needed to understand factors that influence SB following surgery and to make reducing SB and engaging with mobile health technology after surgery more feasible and beneficial for these high-risk patients.

## Appendix

Multimedia Appendix 1 CONSORT-eHEALTH checklist (V 1.6.2).

## Abbreviations

DASH: Detecting Activity to Support Healing
SB: sedentary behavior

## Data Availability

Data generated and analyzed during this study are not publicly available to protect patient health information but are available as deidentified data from the corresponding author on reasonable request.
