# Supplementary material for: A Real-Time Mobile Intervention to Reduce Sedentary Behavior Before and After Cancer Surgery: Pilot Randomized Controlled Trial
Source: JMIR Perioper Med. 2023 Jan 12;6:e41425. doi: 10.2196/41425 (PMC9880805; doi:10.2196/41425)
Supplement: Multimedia Appendix 1 [file periop_v6i1e41425_app1.pdf]

| CONSORT-EHEALTH Checklist V1.6.2 Report | Manuscript Number | 41425 |
|---|---|---|
| **(based on CONSORT-EHEALTH V1.6), available at [http://tinyurl.com/consort-ehealth-v1-6].** | | |
| | | |
| **Date completed** | | |
| 1/3/2023 14:29:37 | | |
| **by** | | |
| Carissa Low | | |
| | | |
| A Real-Time Mobile Intervention to Reduce Sedentary Behavior Before and After Cancer Surgery: Pilot Randomized Trial | | |
| TITLE | | |
| **1a-i) Identify the mode of delivery in the title** | | |
| "A Real-Time Mobile Intervention to Reduce Sedentary Behavior Before and After Cancer Surgery: Pilot Randomized Trial" | | |
| **1a-ii) Non-web-based components or important co-interventions in title** | | |
| not applicable - intervention was exclusively delivered via mobile technology | | |
| **1a-iii) Primary condition or target group in the title** | | |
| "A Real-Time Mobile Intervention to Reduce Sedentary Behavior Before and After Cancer Surgery: Pilot Randomized Trial" | | |
| ABSTRACT | | |
| **1b-i) Key features/functionalities/components of the intervention and comparator in the METHODS section of the ABSTRACT** | | |
| "Patients scheduled for surgery for metastatic gastrointestinal cancer (n=6) were enrolled and randomized to receive either the SB intervention or activity monitoring only. Both groups used a Fitbit smartwatch and companion smartphone app to rate daily symptoms and collect continuous objective activity behavior data starting from at least 10 days before surgery through 30 days post discharge. Participants in the intervention group also received prompts to walk after any SB bout that exceeded a prespecified threshold, with less frequent prompts on days that patients reported more severe symptoms." | | |
| **1b-ii) Level of human involvement in the METHODS section of the ABSTRACT** | | |
| not applicable -- no human involvement in intervention | | |
| **1b-iii) Open vs. closed, web-based (self-assessment) vs. face-to-face assessments in the METHODS section of the ABSTRACT** | | |
| "Participants completed end-of-study ratings of acceptability, and we also examined adherence to assessments and to walking prompts. In addition, we examined effects of the intervention on objective SB and step counts, patient-reported quality of life and depressive and physical symptoms, as well as readmissions." | | |
| **1b-iv) RESULTS section in abstract must contain use data** | | |
| "Accrual (74%), retention (88%), and acceptability ratings (mean overall satisfaction 88.5/100, SD 9.1) were relatively high. However, adherence to assessments and engagement with the SB intervention decreased significantly after surgery and did not recover to preoperative levels after postoperative discharge." | | |
| **1b-v) CONCLUSIONS/DISCUSSION in abstract for negative trials** | | |
| "Perioperative patients with metastatic gastrointestinal cancer were interested in a real-time SB intervention and rated the intervention as highly acceptable, but engagement with the intervention and with daily symptom and activity monitoring decreased significantly after surgery. There were no significant effects of the intervention on step counts, patient-reported quality of life or symptoms, and postoperative readmissions, and there was an apparent adverse effect on maximum SB. " | | |
| INTRODUCTION | | |
| **2a-i) Problem and the type of system/solution** | | |
| "Surgical treatment is a critical component of curative therapy for most cancers, but risks for postoperative complications, unplanned readmissions, and persistent functional impairments are common, especially for abdominal cancers, where rates of complications and readmissions can range from 25%-50% [1-3]. These high rates of adverse postoperative outcomes place patients at risk for functional limitations and impaired quality of life as well as high health care costs and utilization. Supportive interventions aimed at optimizing perioperative health and functioning are needed for this high-risk surgical oncology population." | | |
| **2a-ii) Scientific background, rationale: What is known about the (type of) system** | | |
| "Physical activity is one modifiable behavior that holds promise for affecting postoperative recovery and outcomes [4-6]. Indeed, prehabilitation programs that promote physical activity before surgery have been linked to improved preoperative functional capacity [7] and shorter length of stay after cancer surgery [8]. Similarly, early mobilization after surgery, generally defined as out of bed activity by postoperative day one, is recommended as part of Enhanced Recovery after Surgery pathways, although evidence of benefit is mixed [9]. Because both prehabilitation and Enhanced Recovery after Surgery often include nutritional interventions and other components, it is difficult to determine whether and to what extent increased physical activity alone can reduce postoperative risks. Moreover, postoperative symptoms such as pain and fatigue make increasing physical activity after cancer surgery challenging and may compromise adherence to exercise interventions [10,11]. In the perioperative surgical oncology context, interventions aimed at disrupting prolonged sedentary behavior (SB) with brief walking breaks may be more attainable than more structured exercise interventions, especially if the intervention can adapt to changing symptom burden over the perioperative course. To date, no studies have tested the impact of perioperative SB disruption on surgical oncology outcomes [12]." | | |
| **Does your paper address CONSORT subitem 2b?** | | |
| "The goal of this study was to pilot-test a personalized mobile technology–supported intervention to reduce SB before and after gastrointestinal cancer surgery." | | |
| METHODS | | |
| **3a) CONSORT: Description of trial design (such as parallel, factorial) including allocation ratio** | | |
| "Following completion of written informed consent, participants were randomized via random number generator to either the SB intervention (which included activity monitoring) or activity monitoring only." | | |
| **3b) CONSORT: Important changes to methods after trial commencement (such as eligibility criteria), with reasons** | | |
| not applicable to the current study | | |
| **3b-i) Bug fixes, Downtimes, Content Changes** | | |
| not applicable to the current study | | |
| **4a) CONSORT: Eligibility criteria for participants** | | |
| "The study was open to English-speaking adults scheduled for surgical treatment of metastatic gastrointestinal or peritoneal cancer and able to stand and walk unassisted. Exclusion criteria included having less than ten days to scheduled surgery date, to provide adequate time for participants to become familiar with study technology and activity prompts prior to surgery. No participants had sensory or motor impairments that interfered with use of the study apps." | | |
| **4a-i) Computer / Internet literacy** | | |
| "Owns a smartphone, n (%)26 (100)12 (100)13 (100)" | | |
| **4a-ii) Open vs. closed, web-based vs. face-to-face assessments:** | | |
| "Participants were recruited from the outpatient clinics of 6 surgeons specializing in abdominal cancer surgery at a National Cancer Institute–designated comprehensive cancer center. Participants were recruited between June 2019 and March 2021 at their preoperative surgical oncology clinic visit. Study accrual was paused from March to December of 2020 due to the COVID-19 pandemic." | | |
| **4a-iii) Information giving during recruitment** | | |
| "Research staff provided study information and email reminders to the 6 surgical oncology care teams and asked the nurse or physician assistant to identify potential patients at the time of their consent to surgery, confirm their eligibility, and to either consent them directly or connect them with the research team for consent and onboarding." | | |
| **4b) CONSORT: Settings and locations where the data were collected** | | |
| "Participants were recruited from the outpatient clinics of 6 surgeons specializing in abdominal cancer surgery at a National Cancer Institute–designated comprehensive cancer center." | | |
| **4b-i) Report if outcomes were (self-)assessed through online questionnaires** | | |
| "Participants completed a questionnaire at study entry to collect information about demographic variables, health behaviors, and experience with mobile technology. Before surgery, during inpatient recovery, and approximately 30 days after postoperative discharge, participants also completed standardized measures of depressive and physical symptoms and quality of life. At the end of the study, all participants completed a semistructured interview about their experiences with the devices and a questionnaire about the acceptability and usability of the apps." | | |
| **4b-ii) Report how institutional affiliations are displayed** | | |
| not applicable to the current study | | |
| **5) CONSORT: Describe the interventions for each group with sufficient details to allow replication, including how and when they were actually administered** | | |
| **5-i) Mention names, credential, affiliations of the developers, sponsors, and owners** | | |
| Described in previous publication [reference 13] | | |
| **5-ii) Describe the history/development process** | | |
| Described in previous publication [reference 13] | | |
| **5-iii) Revisions and updating** | | |
| Described in previous publication [reference 13] | | |

| | | |
|---|---|---|
| **5-iv) Quality assurance methods** | | |
| Described in previous publication [reference 13] | | |
| **5-v) Ensure replicability by publishing the source code, and/or providing screenshots/screen-capture video, and/or providing flowcharts of the algorithms used** | | |
| Described in previous publication [reference 13] | | |
| **5-vi) Digital preservation** | | |
| Described in previous publication [reference 13] | | |
| **5-vii) Access** | | |
| Described in previous publication [reference 13] | | |
| **5-viii) Mode of delivery, features/functionalities/components of the intervention and comparator, and the theoretical framework** | | |
| "As previously described [13], all participants used the DASH Android smartphone study app to rate the daily severity of 10 symptoms (ie, pain, fatigue or tiredness, sleep disturbance, trouble concentrating or remembering things, feeling sad or down, feeling anxious or worried, shortness of breath, numbness or tingling, nausea, and diarrhea or constipation), using a scale from 0 (ie, symptom not present) to 10 (ie, symptom as bad as you can imagine). Participants were randomized to either the DASH intervention or monitoring-only control condition. Participants randomized to the DASH intervention received a Fitbit smartwatch app that used the most recent symptom rating to set a threshold for SB bouts and used real-time step count data to trigger activity prompt notifications when prespecified SB thresholds were exceeded (60 consecutive minutes of SB when all symptoms were rated less than 7 out of 10 or 120 consecutive minutes of SB if any symptom was rated as 7 or higher). For the purposes of this study, SB was operationalized as a minute with fewer than 10 steps logged by the Fitbit, to allow for incidental stepping and arm movements that might be misclassified as steps, while also classifying very slow walking as activity, given the perioperative context and likely diminished gait cadence during early postoperative recovery [14]. When SB thresholds were exceeded, an activity prompt ("Ready for a short walk?") was sent to the smartwatch. If 30 or more steps were logged within 15 minutes of an activity prompt, participants received a positive feedback message ("Great job being active!"). Prompts were sent only between each participant's waking time and bedtime, which were set by participants in the Android app and could be adjusted during the study. Participants randomized to monitoring-only received a Fitbit smartwatch app that measured steps but did not send activity prompts." | | |
| **5-ix) Describe use parameters** | | |
| "From the time of consent to 30 days after hospital discharge following their surgery, participants were asked to keep the devices charged, to wear the smartwatch as much as possible, to rate their daily experience of symptom severity once each morning, and for intervention participants only, to respond to activity prompts." | | |
| **5-x) Clarify the level of human involvement** | | |
| not applicable - no human involvement | | |
| **5-xi) Report any prompts/reminders used** | | |
| "When SB thresholds were exceeded, an activity prompt ("Ready for a short walk?") was sent to the smartwatch. If 30 or more steps were logged within 15 minutes of an activity prompt, participants received a positive feedback message ("Great job being active!"). Prompts were sent only between each participant's waking time and bedtime, which were set by participants in the Android app and could be adjusted during the study" | | |
| **5-xii) Describe any co-interventions (incl. training/support)** | | |
| not applicable - no co-interventions in the present study | | |
| **6a) CONSORT: Completely defined pre-specified primary and secondary outcome measures, including how and when they were assessed** | | |
| "Primary outcome measures assessing feasibility were (1) accrual and retention rates (ie, percentage of participants approached who enrolled in the research and percentage of participants enrolled who completed the study); (2) acceptability, based on end-of-study responses to the System Usability Scale [15] and the following questions: "On a scale of 0-100, how easy was it to use the smartphone/watch?" "On a scale of 0-100, how pleasant was the smartphone/watch interface (appearance, design, usability)?" and "On a scale of 0-100, how satisfied were you with the overall system (including the smartphone and watch and all notifications)?"; as well as (3) adherence (percentage of days symptom ratings were completed and at least 8 hours of Fitbit data were logged, and for intervention participants only, percentage of activity prompts after which steps were detected)." | | |
| **6a-i) Online questionnaires: describe if they were validated for online use and apply CHERRIES items to describe how the questionnaires were designed/deployed** | | |
| not relevant to the current study | | |
| **6a-ii) Describe whether and how "use" (including intensity of use/dosage) was defined/measured/monitored** | | |
| "adherence (percentage of days symptom ratings were completed and at least 8 hours of Fitbit data were logged, and for intervention participants only, percentage of activity prompts after which steps were detected" | | |
| **6a-iii) Describe whether, how, and when qualitative feedback from participants was obtained** | | |
| "When asked what they thought of the study, participants in the intervention condition reported that the activity prompts were motivating, especially prior to surgery. For example, one participant (P13) noted that "it got me moving more than I normally would have." However, participants also noted that the prompts were not as motivating or as easy to respond to after surgery, especially in the hospital." | | |
| **6b) CONSORT: Any changes to trial outcomes after the trial commenced, with reasons** | | |
| "Participants were recruited from the outpatient clinics of 6 surgeons specializing in abdominal cancer surgery at a National Cancer Institute–designated comprehensive cancer center." | | |
| **7a) CONSORT: How sample size was determined** | | |
| **7a-i) Describe whether and how expected attrition was taken into account when calculating the sample size** | | |
| not applicable to the current study | | |
| **7b) CONSORT: When applicable, explanation of any interim analyses and stopping guidelines** | | |
| "Primary outcome measures assessing feasibility were (1) accrual and retention rates (ie, percentage of participants approached who enrolled in the research and percentage of participants enrolled who completed the study); (2) acceptability, based on end-of-study responses to the System Usability Scale [15] and the following questions: "On a scale of 0-100, how easy was it to use the smartphone/watch?" "On a scale of 0-100, how pleasant was the smartphone/watch interface (appearance, design, usability)?" and "On a scale of 0-100, how satisfied were you with the overall system (including the smartphone and watch and all notifications)?"; as well as (3) adherence (percentage of days symptom ratings were completed and at least 8 hours of Fitbit data were logged, and for intervention participants only, percentage of activity prompts after which steps were detected)." | | |
| **8a) CONSORT: Method used to generate the random allocation sequence** | | |
| "Following completion of written informed consent, participants were randomized via random number generator to either the SB intervention (which included activity monitoring) or activity monitoring only. " | | |
| **8b) CONSORT: Type of randomisation; details of any restriction (such as blocking and block size)** | | |
| "Following completion of written informed consent, participants were randomized via random number generator to either the SB intervention (which included activity monitoring) or activity monitoring only. " | | |
| **9) CONSORT: Mechanism used to implement the random allocation sequence (such as sequentially numbered containers), describing any steps taken to conceal the sequence until interventions were assigned** | | |
| "Following completion of written informed consent, participants were randomized via random number generator to either the SB intervention (which included activity monitoring) or activity monitoring only. " | | |
| **10) CONSORT: Who generated the random allocation sequence, who enrolled participants, and who assigned participants to interventions** | | |
| "Research staff provided study information and email reminders to the 6 surgical oncology care teams and asked the nurse or physician assistant to identify potential patients at the time of their consent to surgery, confirm their eligibility, and to either consent them directly or connect them with the research team for consent and onboarding." | | |
| **11a) CONSORT: Blinding - If done, who was blinded after assignment to interventions (for example, participants, care providers, those assessing outcomes) and how** | | |
| **11a-i) Specify who was blinded, and who wasn't** | | |
| not relevant to the current study | | |
| **11a-ii) Discuss e.g., whether participants knew which intervention was the "intervention of interest" and which one was the "comparator"** | | |
| Participants were not told which intervention was the intervention of interest or the comparator | | |
| **11b) CONSORT: If relevant, description of the similarity of interventions** | | |
| Interventions were identical except for the prompts to walk in the Intervention condition | | |
| **12a) CONSORT: Statistical methods used to compare groups for primary and secondary outcomes** | | |
| "Linear mixed modeling assuming the best-fitting variance-covariance structure for the repeated assessments was used to explore the effect of the intervention over the 3 study time points (ie, preoperative, inpatient, and after discharge) for the outcomes of adherence, SB, physical activity, psychological and physical symptoms, and quality of life." | | |
| **12a-i) Imputation techniques to deal with attrition / missing values** | | |
| Not relevant to the current study | | |
| **12b) CONSORT: Methods for additional analyses, such as subgroup analyses and adjusted analyses** | | |
| Not reelvant to the current study | | |
| RESULTS | | |
| **13a) CONSORT: For each group, the numbers of participants who were randomly assigned, received intended treatment, and were analysed for the primary outcome** | | |
| See Table 1 for this information | | |
| **13b) CONSORT: For each group, losses and exclusions after randomisation, together with reasons** | | |

| | | |
|---|---|---|
| "The retention rate for the study was 88%, with 3 participants withdrawing (2 participants before starting to use the devices (one in each condition) and 1 participant in the intervention condition 18 days after surgery due to poor health and readmission)." | | |
| **13b-i) Attrition diagram** | | |
| "The retention rate for the study was 88%, with 3 participants withdrawing (2 participants before starting to use the devices (one in each condition) and 1 participant in the intervention condition 18 days after surgery due to poor health and readmission)." | | |
| **14a) CONSORT: Dates defining the periods of recruitment and follow-up** | | |
| "Participants were recruited between June 2019 and March 2021 at their preoperative surgical oncology clinic visit." | | |
| **14a-i) Indicate if critical "secular events" fell into the study period** | | |
| not applicable to the current study | | |
| **14b) CONSORT: Why the trial ended or was stopped (early)** | | |
| "Study accrual was paused from March to December of 2020 due to the COVID-19 pandemic." | | |
| **15) CONSORT: A table showing baseline demographic and clinical characteristics for each group** | | |
| See Table 1 for this information | | |
| **15-i) Report demographics associated with digital divide issues** | | |
| See Table 1 for this information | | |
| **16a) CONSORT: For each group, number of participants (denominator) included in each analysis and whether the analysis was by original assigned groups** | | |
| **16-i) Report multiple "denominators" and provide definitions** | | |
| See Table 1 for this information | | |
| **16-ii) Primary analysis should be intent-to-treat** | | |
| Primary analysis were intent-to-treat | | |
| **17a) CONSORT: For each primary and secondary outcome, results for each group, and the estimated effect size and its precision (such as 95% confidence interval)** | | |
| See Results section, including Figures 1-3 | | |
| **17a-i) Presentation of process outcomes such as metrics of use and intensity of use** | | |
| "Over the course of the study, daily symptom ratings were completed on 62% (874/1416) of days, ranging from 14% (9/65) to 95% (55/58) of days across individual participants. Fitbits were worn on 77% (1091/1416) of study days, and 91.7% (990/1091) of these days had at least 8 hours of Fitbit data available. On average, 69% (977/1416) of days were included in Fitbit analyses; across individual participants, the percentage of days with at least 8 hours of Fitbit data ranged from 15% (9/52 days with ≥8 hours of data) to 100% (58/58 days with ≥8 hours of data). As shown in Figure 1, participants became less adherent with both symptom reporting and wearing the Fitbit after surgery (symptom reporting: $F_{time}$=22.9, P<.0001; Fitbit: $F_{time}$=9.2, P=.001), but there were no significant differences in adherence between the two study groups (symptom reporting: $F_{group}$=0.0, P=.950 and $F_{group×time}$=0.3, P=.78; Fitbit: $F_{group}$=0.2, P=.663 and $F_{group×time}$=0.4, P=.68)." | | |
| **17b) CONSORT: For binary outcomes, presentation of both absolute and relative effect sizes is recommended** | | |
| Not applicable to the current study | | |
| **18) CONSORT: Results of any other analyses performed, including subgroup analyses and adjusted analyses, distinguishing pre-specified from exploratory** | | |
| Not applicable to the current study | | |
| **18-i) Subgroup analysis of comparing only users** | | |
| Not applicable to the current study | | |
| **19) CONSORT: All important harms or unintended effects in each group** | | |
| "Contrary to hypotheses, participants randomized to the intervention group exhibited longer maximum SB bouts relative to the monitoring-only condition." | | |
| **19-i) Include privacy breaches, technical problems** | | |
| Not applicable to the current study | | |
| **19-ii) Include qualitative feedback from participants or observations from staff/researchers** | | |
| Qualitative feedback is reported in Results | | |
| DISCUSSION | | |
| **20) CONSORT: Trial limitations, addressing sources of potential bias, imprecision, multiplicity of analyses** | | |
| **20-i) Typical limitations in ehealth trials** | | |
| "This study had a number of important limitations. First, the sample size was smaller than originally intended due to COVID-19 pandemic–related disruptions to accrual and is an important limitation of this study. Second, developing a system capable of remotely detecting real-time step counts using a consumer wearable device proved challenging and required us to provide study Android phones to participants to use for collecting symptom ratings and synchronizing the Fitbit smartwatch. All enrolled participants already owned a personal smartphone; requiring them to also carry and charge an additional study smartphone across perioperative transitions of care may have contributed to adherence challenges; future interventions in this area should be deployed on participants' existing phones to potentially improve feasibility. Third, we elected to use a monitoring-only control so that the only difference between the two study groups was the activity prompts in recognition of the fact that merely using an activity monitor can promote physical activity among cancer patients [25]; alternative control conditions could have yielded different results. Finally, all participants were undergoing surgery for metastatic peritoneal or gastrointestinal cancer, and results may not generalize to other perioperative groups or contexts." | | |
| **21) CONSORT: Generalisability (external validity, applicability) of the trial findings** | | |
| **21-i) Generalizability to other populations** | | |
| "Finally, all participants were undergoing surgery for metastatic peritoneal or gastrointestinal cancer, and results may not generalize to other perioperative groups or contexts." | | |
| **21-ii) Discuss if there were elements in the RCT that would be different in a routine application setting** | | |
| not applicable to current study | | |
| **22) CONSORT: Interpretation consistent with results, balancing benefits and harms, and considering other relevant evidence** | | |
| **22-i) Restate study questions and summarize the answers suggested by the data, starting with primary outcomes and process outcomes (use)** | | |
| "In this paper, we described results from a pilot randomized trial testing a SB intervention in patients undergoing surgery for metastatic gastrointestinal cancer. To our knowledge, this is the first SB intervention developed specifically for patients with surgical oncology at high risk for adverse postoperative outcomes [12]. Although patients were willing to participate and remain in the trial and rated the intervention as highly acceptable, engagement with the intervention and with daily symptom and activity monitoring decreased significantly after surgery. There were no significant effects of the intervention on step counts, patient-reported quality of life or symptoms, and postoperative readmissions. Contrary to hypotheses, participants randomized to the intervention group exhibited longer maximum SB bouts relative to the monitoring-only condition." | | |
| **22-ii) Highlight unanswered new questions, suggest future research** | | |
| "The SB intervention tested in this trial was designed to make replacing prolonged SB with short walking breaks more feasible in the perioperative context by reducing the frequency of SB prompts on days that patients reported high symptom burden. Given the significant drops in adherence and engagement that occurred after surgery and particularly during inpatient recovery as well as the fact that participants may have been less likely to complete symptom ratings on days they felt particularly unwell, additional modifications to the intervention are needed to make postoperative activity more feasible. A number of participants in the intervention condition noted that it was very difficult to get out of bed to walk when prompted, especially without assistance; this challenge and the associated frustration could have led to decreased self-efficacy to adhere to the intervention that carried into the postdischarge period, leading to increased SB and decreased adherence. Either pausing the intervention until patients were recovering at home, adapting the intervention to involve caregivers or hospital staff and timing prompts around their availability to assist with postoperative ambulation, or replacing walking with light stretches or activities that could be done in bed while seated could be options for future interventions targeting activity among postoperative inpatients. Adherence to assessments also decreased in the monitoring-only group after surgery, suggesting that either reduced frequency of assessments or additional support and reminders may be needed to make collection of continuous activity and daily symptom ratings feasible postoperatively.<br><br>The lack of observed benefits with regard to SB and activity may have been related to low adherence and engagement, or the intervention may not have been sufficiently robust to produce a change in activity during the acute perioperative period. Although participants in our usability and feasibility study reported that the frequency of prompts was appropriate, the current intervention was fairly minimal, aiming to disrupt SB bouts of 1-2 hours with a small (30 steps or more) amount of walking and positive reinforcement when goals were met. Participants may have disengaged from the intervention if they did not perceive it to be beneficial, and targeting a higher activity goal, if done in a feasible way that factors in physical limitations after surgery, could result in higher adherence over time if participants perceive the SB intervention to be meaningfully increasing activity and to have potential health benefits. In the future, providing education about the risks of perioperative SB, personalized goal setting to inform prompts, and coaching and problem-solving to overcome barriers to disrupting SB could be considered as additional interventional components to enhance a perioperative SB intervention [20,12]. Involving patients in the co-design of perioperative SB interventions could also result in enhanced engagement and benefit [21]." | | |
| Other information | | |
| **23) CONSORT: Registration number and name of trial registry** | | |
| "registered on ClinicalTrials.gov (NCT03211806)" | | |
| **24) CONSORT: Where the full trial protocol can be accessed, if available** | | |
| not applicable to the current study | | |
| **25) CONSORT: Sources of funding and other support (such as supply of drugs), role of funders** | | |
| Provided under Acknowledgements | | |
| **X26-i) Comment on ethics committee approval** | | |

| | | |
|---|---|---|
| All procedures were approved by the University of Pittsburgh Institutional Review Board (STUDY 19030389) and registered on ClinicalTrials.gov (NCT03211806). | | |
| **x26-ii) Outline informed consent procedures** | | |
| "Research staff provided study information and email reminders to the 6 surgical oncology care teams and asked the nurse or physician assistant to identify potential patients at the time of their consent to surgery, confirm their eligibility, and to either consent them directly or connect them with the research team for consent and onboarding. " | | |
| **X26-iii) Safety and security procedures** | | |
| not relevant to the current study | | |
| **X27-i) State the relation of the study team towards the system being evaluated** | | |
| Reported in previous publication [13] | | |
